# The Correlation Analysis of Microstructure and Tribological Characteristics of In Situ VCp Reinforced Iron-Based Composite

**DOI:** 10.3390/ma14154343

**Published:** 2021-08-03

**Authors:** Yun Zhang, Richen Lai, Qiang Chen, Zhen Liu, Ruiqing Li, Jufei Chen, Pinghu Chen

**Affiliations:** 1 State Key Laboratory of High Performance Complex Manufacturing, Light Alloys Research Institute, College of Mechanical and Electrical Engineering, Central South University, Changsha 410083, China; yun_zhang66@163.com (Y.Z.); liruiqing@csu.edu.cn (R.L.); chenjufei1988@163.com (J.C.); 2 College of Mechatronics & Control Engineering, Shenzhen University, Shenzhen 518060, China; 1910293034@email.szu.edu.cn (R.L.); 1910293020@email.szu.edu.cn (Q.C.); liuzhen598352368@126.com (Z.L.)

**Keywords:** in situ vanadium carbide, wear-resistant composite, heat treatment, phase transformation, mechanical properties, tribological behavior

## Abstract

In this study, four kinds of heat treatments were performed to obtain a certain amount of retained austenite, which can result in good toughness and low brittleness accompanied with wear resistance of an in situ VC particle reinforced iron-based composite (VCFC). Microstructure, mechanical properties and wear resistance of the samples under heat treatment of QP, QPT, MQP and MQPT were compared. The experimental results indicated that there is a huge difference in microstructure between MQPT and the other heat treatments. High-proportion retained austenite and white net-like precipitates of M_7_C_3_ carbide existed in the MQPT-treated sample, but thick M_7_C_3_ carbide with brittleness was discovered in the other sample. Thereby, high-proportion retained austenite contributed to its low hardness of 634 HV and high tensile strength of 267 MPa, while a maximum hardness of 705.5 HV and a minimum tensile strength of 205 MPa were exhibited in the QPT-treated sample with a V-rich carbide of high hardness, a Cr-rich carbide of brittleness and a high-proportion martensite. Meanwhile, a phase transformation from retained austenite to martensite could increase the hardness and enhance wear resistance based on the transformation-induced plasticity (TRIP) effect; its wear rate was only 1.83 × 10^−6^ mm^−3^/(N·m). However, the wear rates of the samples under QP, QPT and MQP heat treatments increased by 16.4%, 44.3% and 41.0%, respectively. The wear mechanism was a synergistic effect of the adhesive wear mechanism and the abrasive wear mechanism. The adhesive wear mechanism was mainly considered in the MQPT-treated sample to reduce the wear rate attributed to high-proportion retained austenite and the existence of wear debris with a W element on the surface of the wear track. However, the abrasive wear mechanism could exist in the other samples because of a lot of thick, brittle M_7_C_3_, thereby resulting in a higher wear rate due to immediate contact between the designed material and the counterpart.

## 1. Introduction

The iron-based composites reinforced with hard ceramic particles (carbides, nitrides, metallic oxides, intermetallic compounds, etc.), which possess an excellent hardness and wear resistance, have been applied in the automobile, aerospace, mining, metallurgy and civil engineering industries [1,2,3,4,5]. In situ VC particle reinforced iron-based composites were developed due to spontaneous growth of vanadium carbide during solidification [6,7]. In situ VC particles are uniformly distributed in the iron matrix accompanied with their good metallurgical bonding, which results in good hardness and wear resistance [8]. In order to obtain more excellent wear resistance, a large amount of VC particles should be provided by adding a large amount of V and C elements [9,10], resulting in low toughness, high fatigue damage or an initial failure [11]; this limits the wide application of our designed material for serious impact and high wear components (wear-resistant elbow, milling cutter, washer, etc.) in engineering equipment. To solve this problem, the corresponding heat treatments were performed to improve the toughness because of the formation of retained austenite with a certain ratio [12,13], but the trade-offs of wear resistance and toughness were not realized, thus resulting in the limits of its bright applied future.

Transformation-induced plasticity (TRIP) aided steel has a comprehensive performance with high strength and excellent toughness because of an amount of metastable austenite [14,15], and it can fulfill the purpose of wear resistance-toughness trade-offs by the addition of ceramic particles and post heat treatment [16,17]. The role of retained austenite on wear resistance of nanostructured carbide-free bainitic steel was discussed by P. V. Moghaddam; the results indicated that better wear resistance was obtained because a higher work hardening capacity was increased with a high TRIP effect [18]. In addition, Q&P heat treatment was carried out to obtain the retained austenite, which contributes to high strength properties, and excellent wear resistance was caused by phase transformation of retained austenite [19]. In our previous works, a novel in situ VCp reinforced iron-based composite (VCFC) was designed based on the transformation-induced plasticity (TRIP) effect. Simultaneously, the corresponding heat treatment was performed to obtain the retained austenite (FCC) of metastable structure, which was then transformated from an FCC structure of good toughness to a BCC structure of high hardness under a certain load [20,21,22]. Previous results indicate that Mn-partition and C-partition can stabilize the retained austenite at room temperature accompanied with excellent wear resistance. However, the wear resistance of the samples with different heat treatments was not studied systematically in our previous works.

In this work, tempering treatment was carried out to tailor the microstructure, mechanical properties and wear resistance based on heat treatment of quenching–partitioning (QP) and Manganese partitioning–quenching–partitioning (MQP) to assess the influence of heat treatment on the microstructure, mechanical properties and wear resistance. In particular, the wear mechanism was explored in detail for different friction conditions.

## 2. Experimental Procedure

A VCFC was prepared by a 200 kg medium frequency electromagnetic induction furnace (HJZ-300KW, Hengjia, Dongguan, China). The chemical composition mainly included 8.1 V, 3.0 Mn, 2.8 C, 2.5 Cr, 1.5 Mo, 1.5 Ti, 1.5 Si, bal. Fe (wt.%). In order to obtain uniform distribution of all solute elements in the molten iron: Firstly, pig iron was melted in an electromagnetic induction furnace, and the slag floating on the surface of the molten iron was removed. Secondly, ferromanganese iron, ferrochromium, ferromolybdenum and ferrovanadium were gradually added into the molten iron, and then deoxidization was carried out by adding a certain amount of aluminum element. Thirdly, a titanium element was added and considered as a heterogeneous nucleating element. The temperature of the molten iron was adjusted at 1500 °C for a period of time. The molten iron with 1450 °C was poured into the sand system with 200 mm × 100 mm × 20 mm; finally, all ingots were cooled to 200 °C in the casting sand and then the air was cooled to room temperature. Afterwards, a wire-cut electric discharge machine was adopted to cut heat-treated specimens of 50 mm × 30 mm × 10 mm. Four kinds of heat treatment techniques were performed and the detailed process is presented in Table 1. After heat treatment, the samples were cut for microstructural characterization, microhardness testing, tensile measurement and friction-wear testing.

All samples for microhardness, tensile strength and wear were prepared by mechanical grinding using 400, 800, 1200 and 1500 grit SiC papers. Firstly, the digital micro Vickers hardness tester (Micro Vickers HV-1000Z, MEGA INSTRUMENTS, Shanghai, China) was employed to measure the microhardness of samples with different heat treatments under a load of 1000 gf with a loading duration time of 15 s; each hardness value was obtained by on average at least five points, resulting in reaching the final value. Secondly, tensile strength was measured using universal testing machines (PWS-E100, Shenggong, Jinan, China) the detailed sizes of the samples and testing parameters have been reported in our previous work [22]. Thirdly, the friction and wear tests were carried out using a ball-on-disk machine (MFT-5000, Rtec Instrument, San Jose, CA, USA) with a φ 4 mm tungsten carbide ceramic ball as the counterpart under different wear loads of 5, 10, 20 N and different wear times of 1, 3, 5 h; meanwhile, the turning speed and radius were set to 200 r/min and 5 mm, respectively. Afterwards, 3D laser confocal scanning microscopy (VK-X200 series, Keyence Corporation of America, Itasca, IL, USA) was performed to characterize the morphology of the wear tracks and to calculate wear rate according to the equation w = V/(L·F); w is wear rate, mm^3^/(m·N); V is wear volume, which is equal to 2πr·S_wear_, mm^3^; L represents total travel, which is 2πr·200 r/min·t, m; F is wear load, N. The samples for the friction-wear test were ground by 1500 grit sand paper, but the roughness (Ra) of the surface was still about 3 μm because of the existence of a multi-phase with an iron matrix (relatively soft) and hard carbides such as VC and M_7_C_3_.

Scanning electron microscope (SEM, TESCAN MIRA 3 LMH/LMU, Brno-Kohoutovice, Czech Republic) operated at 20 kV was employed to characterize the shape, size and distribution of the precipitate using the synergetic characterization of back scattered electron (BSE) and secondary electron (SE); the samples were etched with nitrohydrochloric acid (Huihong, Changsha, China) (the ratio of concentrated nitric acid and hydrochloric acid was 3:1) for 20 s. Meanwhile, an energy dispersive spectroscopy (EDS) detector was used to examine elemental distribution. In addition, phase analysis was performed by high-energy X-ray diffraction (HEXRD, Bruker, D8 discover, Karlsruhe, Germany) with a Cu target (λ = 0.15418 nm), which was operated at 45 kV and 200 mA, and the data were collected at 2θ = 20°~100° using a 0.02 step size.

## 3. Results

**Figure 1:**
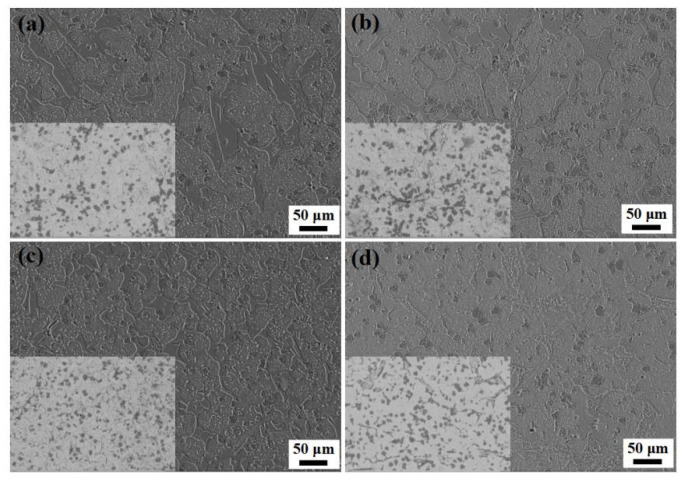
Secondary electron images andbBackscattered electron images of the VCFC with different heat-treated processes. (**a**) QP, (**b**) QPT, (**c**) MQP, (**d**) MQPT.

**Figure 2:**
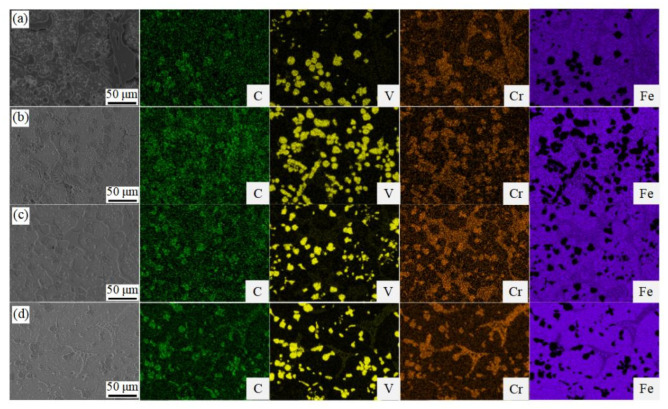
Main alloying elements’ distribution of the VCFC with different heat-treated processes. (**a**) QP, (**b**) QPT, (**c**) MQP, (**d**) MQPT.

The back scattered electron image and the corresponding secondary electron image of the samples with different heat treatments are shown in Figure 1 and Figure 2. In the QP-treated sample, the morphologies mainly include globular-like black particles, irregular thick precipitates with few net-like structure and tiny white precipitates of uniform distribution in the matrix. The uneven distribution of globular-like black particles with the size 10~20 μm is presented in Figure 1a. However, there is a relatively large difference between the QP-treated sample and the other treated samples. For example, black particles are gathered to form flower-like particles when irregular thick precipitates become the thinner and more even distribution in the QPT-treated sample, as shown in Figure 1b. However, uniform distribution of black particles existed in the MQP-treated sample, and the width of irregular thick precipitates decreased accompanied with more consistency, as shown in Figure 1c. Even more specifically, irregular net-like white precipitates were detected without irregular thick precipitates in the MQPT-treated sample, as shown in Figure 1d. Furthermore, according to EDS mapping, black particles are V-rich carbide, and irregular thick precipitates and net-like white precipitates mainly include Fe, Cr and C elements, which can be considered as containing Fe, Cr carbide, as shown in Figure 2.

**Figure 3:**
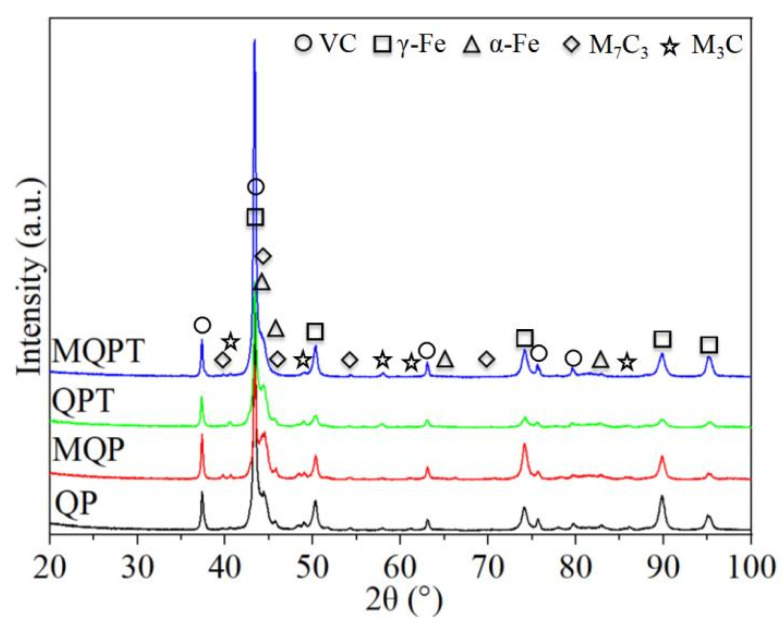
XRD patterns of the VCFC with different heat-treated process.

Combinations with the XRD patterns of the samples after different heat treatments are shown in Figure 3. It can be seen that all the samples were composed of VC, retained austenite of γ-fcc structure, martensite of α-bcc structure, M_7_C_3_ and M_3_C. In this study, the spectra peaks of VC appeared approximately at 2theta = 37.4°, 43.4°, 63.1°, 75.7° and 79.7° for the crystallographic planes of (111), (200), (220), (311) and (222), respectively. In addition, the three strongest peaks of retained austenite appeared at 43.3°, 50.4° and 74.1° for the crystallographic planes of (111), (200) and (220), while the strongest peak of martensite was (110) at 45° approximately. It is also notable that the (110) peak intensity of the MQPT-treated sample decreased while the intensity of four peaks increased relative to the samples under other heat treatments. In addition, the peak intensity of M_7_C_3_ and M_3_C decreased or disappeared in the MQPT-treated sample.

**Figure 4:**
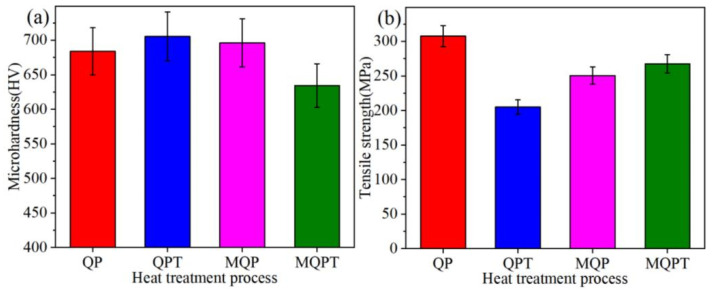
Microhardness (**a**) and tensile strength (**b**) of the VCFC with different heat-treated processes.

**Figure 5:**
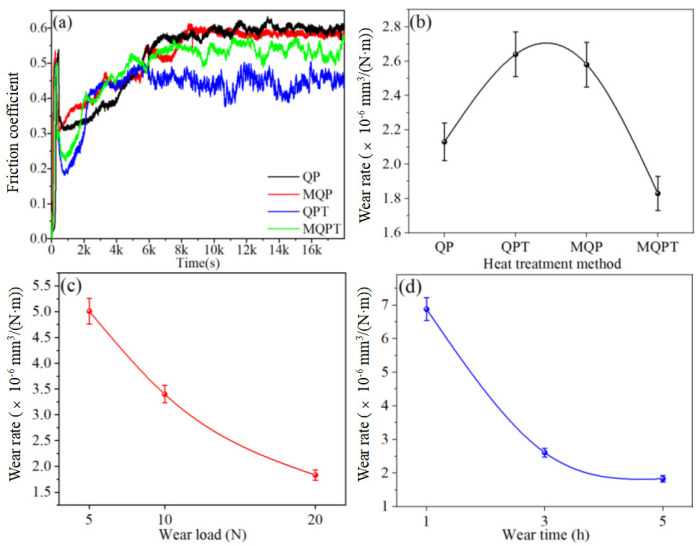
Friction coefficient, microhardness and tensile strength of the VCFC with different heat-treated processes. (**a**) The friction coefficient; (**b**) Wear rate of four specimens under wear load 20 N and wear time 5 h; (**c**) Wear rate of MQPT specimen under wear time 5 h and different wear load; (**d**) Wear rate of MQPT specimen under wear load 20 N and different wear time.

Figure 4 shows the microhardness and tensile strength of the samples with different heat treatments. The microhardness values of QPT and MQP-treated samples were larger than those under QP and MQPT heat treatments. The minimum value was discovered in the sample under MQPT heat treatment, whose value is only 634 HV and 10% lower than that under the QPT-treated sample. However, there was a relatively contradictory trend that emerged in the tensile strength, as shown in Figure 4b. The minimum value emerged in the sample under QPT heat treatment, while the tensile strength of the QP-treated sample reached a maximum value of above 300 MPa. In general, the wear resistance of the sample with larger hardness was better than that with low hardness. However, according to the results shown in Figure 5, the experimental results of friction-wear testing are not in accordance with the common practice. Figure 5a shows the friction coefficient of the samples under different heat treatments; it is obvious that the friction coefficient of the QPT-treated sample is the minimum value, which is about 0.45, while the maximum value of ~0.6 can appear in the sample under QP heat treatment. Meanwhile, the wear rates of four samples with different heat treatments were compared under the conditions of wear load 20 N and wear time 5 h, as shown in Figure 5b; the wear rate of QP, QPT, MQP and MQPT-treated samples first rose then descended; the maximum value of 2.64 × 10^−6^ mm^3^/(N·m) appeared in the sample under QPT heat treatment; at the same time, the wear rate of the MQPT-treated sample had a minimum value of 1.83 × 10^−6^ mm^3^/(N·m). The tribological behaviors of the MQPT-treated sample were emphasized in the analysis under different wear loads and different wear times. Figure 5c indicates that the wear rate gradually decreased with the increase of the wear load—the wear rate under wear load of 5 N is 5.01 × 10^−6^ mm^3^/(N·m)—but the wear rates of 10 and 20 N decreased by 32% and 63%. In addition, the same tendency existed in the wear rate with the increase of wear time; the wear rate reaches 6.88 × 10^−6^ mm^3^/(N·m) when the wear time is 1 h, but the wear rate can be decreased about 3.7 times when the wear time is 5 h.

**Figure 6:**
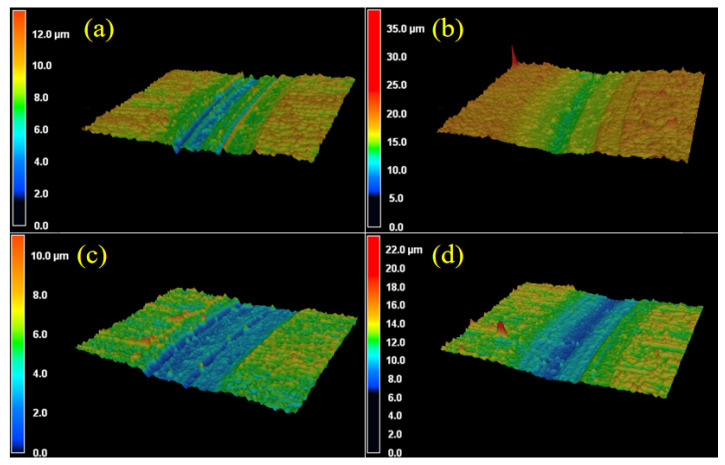
Three-dimensional laser confocal images of the wear tracks with different heat treatment processes under the conditions of wear load 20 N and wear time 5 h. (**a**) QP; (**b**) QPT; (**c**) MQP; (**d**) MQPT.

**Figure 7:**
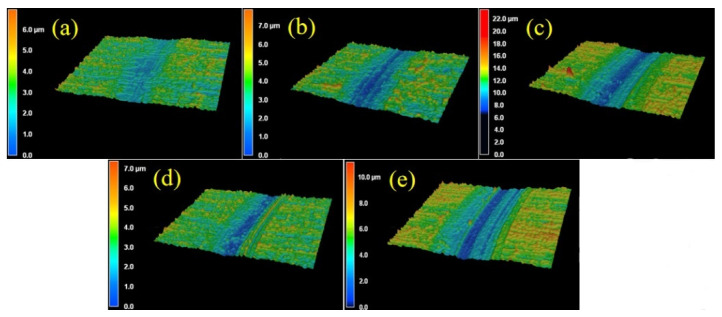
Three-dimensional laser confocal images of the wear tracks of MQPT specimen under different wear loads and wear times. (**a**) 5N, 5h; (**b**) 10N, 5h; (**c**) 20N, 5h; (**d**) 20N, 1h; (**e**) 20N, 3h.

Combined with 3D laser confocal scanning microscopy of the samples under different heat treatments, outline heights were magnified 10 times, as shown in Figure 6 and Figure 7. Figure 6 shows that, for 3D morphology of wear tracks with different heat treatments, the depth of the wear track with the QPT-treated sample is the maximum, and its value can reach about 15 μm under wear load of 20 N and wear time of 5 h, and the deepest region is at the middle of wear track; there is a same phenomenon that appears in the wear track of the MQPT-treated sample, but the depth is only 8 μm and the width is relatively narrow. In the samples of QP and MQP heat treatments, the deepest region is not in the middle of wear tracks but at the edge of wear tracks, as shown in Figure 6a,c; their maximum depth is approximately 10 μm, but the deeper region of the MQP-treated sample is larger than that of the QP-treated sample. Furthermore, the 3D morphology of the wear track for the MQPT-treated sample is discussed emphatically under different wear loads and times, as shown in Figure 7. Due to the low wear load of 5 N, the rough surface still existed at the region of wear track, as shown in Figure 7a, but the rough surface was removed by the counterpart under a wear load of 10 N and multiple deep channels were discovered in the wear track. With the increase of wear load, there was a single channel at the middle of wear track and its depth was larger, as shown in Figure 7c. However, the deep channel was the first to be discovered at the wear time of 1 h for a wear load of 20 N. and the channel became deeper when accompanied with a narrow channel, as shown in Figure 7e, when the wear time was increased to 3 h. Finally, two channels merged to form the single wider channel.

## 4. Discussion

**Figure 8:**
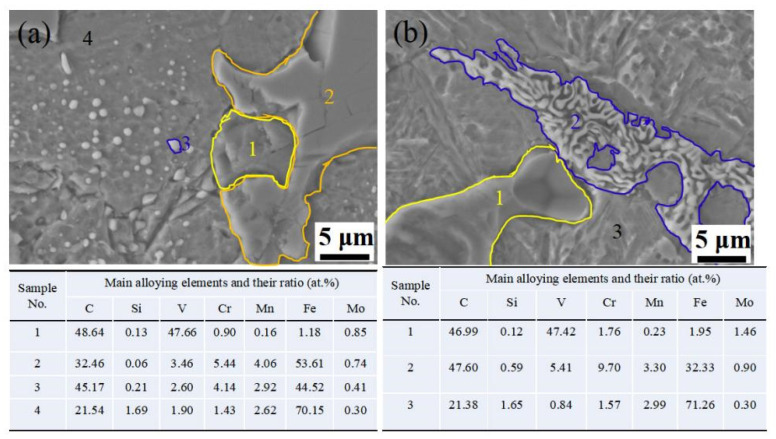
Typical microstructures and their elements ratio under different heat treatment processes. (**a**) QPT; (**b**) MQPT.

VC carbide was in situ formed spontaneously above 1400 °C because of negative Gibbs free energy with △G_0_(kJ/mol) = −102.090 + 0.00958T (298 K < T < 2273 K) [23,24,25], but the low cooling rate of the designed material in clay sand can result in multiple phases, such as M_7_C_3_, M_23_C_6_, M_3_C carbides, iron matrix, etc. [26,27]. Thereby, the microstructure was mainly compared V-rich carbide, Cr-rich carbide and iron matrix of pearlite and martensite structure, but austenite was never discovered in an as-cast sample [20]. However, according to the calculated results of JMatPro software (Sente, Guildford, UK, version 4.0), the austenite ratio has a maximum value at 1261 °C and starts to rapidly decrease until it disappears completely at 697.2 °C. Therefore, the austenitizing temperature was designed and optimized at 1000 °C, followed by quenching at 100 °C, thus resulting in a certain amount of retained austenite at room temperature [21]; Z.C. Li reported that retained austenite was more stabilized when Mn and C elements dissolved into an austenite structure [28]. Therefore, an Mn-partitioning treatment made retained austenite more stable because it suppresses rapid diffusion of alloying elements in the post heat treatment [22]. However, unfortunately, some brittle precipitates were formed and resulted in weakening mechanical properties and wear resistance. In this work, tempering treatment was performed after QP or MQP treatment, and it was noted that tempering treatment with a long time has an important role in the redistribution of M_7_C_3_ or M_3_C, thus resulting in their width being reduced or disappearing. Typical microstructures for QPT and MQPT-treated samples are shown in Figure 8. The same microstructures of QP and MQP-treated samples exist in the QPT-treated sample, and are composed of VC, M_7_C_3_, M_3_C, a large amount of martensite and a certain retained austenite. The existence of VC, M_7_C_3_ and a large amount of martensite contributes to high hardness, as shown in Figure 4a. However, the brittle M_7_C_3_ was easily broken in the process of polishing with glass paper; weakening tensile strength is presented in Figure 4b. By contrast, thick M_7_C_3_ and tiny M_3_C particles were never discovered and net-like M_3_C appeared in the MQPT-treated sample accompanied with a large amount of retained austenite, which can result in low hardness [29], and strain-induced transformation from retained austenite to martensite can occur during the tensile process to improve tensile strength [30], as shown in Figure 4, Figure 5 and Figure 8.

For the results of microhardness and tensile strength, an unusual phenomenon with high hardness and lower tensile strength appeared in the QPT sample. This can be caused by two reasons. On the one hand, a more brittle non-uniform M_7_C_3_ carbide existed in the QPT sample, as shown in Figure 1b. Moreover, as shown in Figure 8a, these brittle carbides were broken under a certain load. On the other hand, the ratio of retained austenite was less than that of other samples, the probability of crack growth increased with a decrease in retained austenite. Therefore, the phenomenon of high hardness and lower tensile strength can occur in the QPT sample.

In addition, wear resistance was enhanced by a synergistic effect between microstructure and the TRIP effect. Generally speaking, excellent wear resistance of the materials was attributed to hardness and friction coefficient, but in this work the hardness of the material was a very important factor. S.Z. Wei et al. noted that morphology features (shape, size and distribution) of the carbides played a dominant role in tribological behaviors when hardness was larger than 58 HRC (655 HV); in contrast, the average hardness of the material was the most significant factor when hardness was less than 58 HRC (655 HV) [31,32]. Concerning tribological features of the samples under different heat treatments in this work, the hardness of QP, MQP and QPT-treated samples were higher than 58 HRC (655 HV) through the conversion between Vickers hardness (HV) and Rockwell hardness (HRC) when the hardness of the MQPT-treated sample was lower than 58 HRC (655 HV), as shown in Figure 4a. Combined with microstructure in Figure 1, Figure 2 and Figure 3, a large amount of VC, M_7_C_3_ and M_3_C carbide existed in the QP, MQP and QPT-treated samples, but brittle M_7_C_3_ carbide was easily broken by pressure from a relatively large load, thus resulting in weak wear resistance and high wear rate. Besides that, an amount of M_7_C_3_ with brittle and high hardness was uniformly distributed in QP and MQP samples. During the process of friction and wear, a serious wear also occurred in counterparts when the tested samples were worn seriously; hard debris existed into wear tracks and was pushed to the edge, followed by what is considered as abrasive particles taking part in the friction and wear, so much so that two channels appeared at the edge of wear tracks for QP and QPT samples. Nevertheless, brittle M_7_C_3_ carbide was never detected in the MQPT-treated sample and Cr, V, Fe-rich net-like precipitates were discovered; while the hardness decreased, wear resistance was enhanced because of the occurrence of martensitic phase transformation.

**Figure 9:**
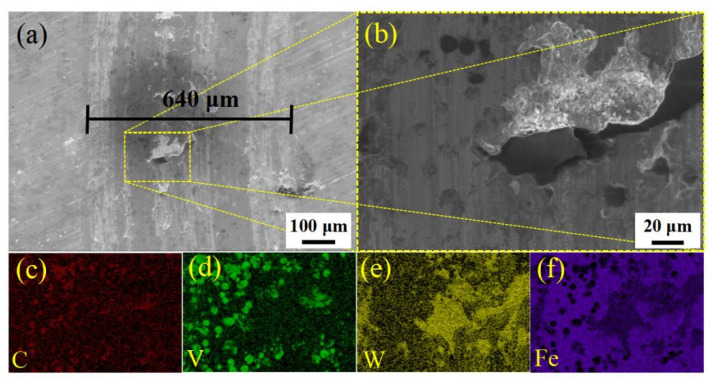
SEM morphologies and the corresponding EDS elemental distribution of wear tracks in the MQPT-treated sample. (**a**) SEM image of wear track; (**b**) Magnified morphology of (**a**) in the yellow box with the dotted line; (**c**) Distribution of C element; (**d**) Distribution of V element; (**e**) Distribution of W element; (**f**) Distribution of Fe element.

**Figure 10:**
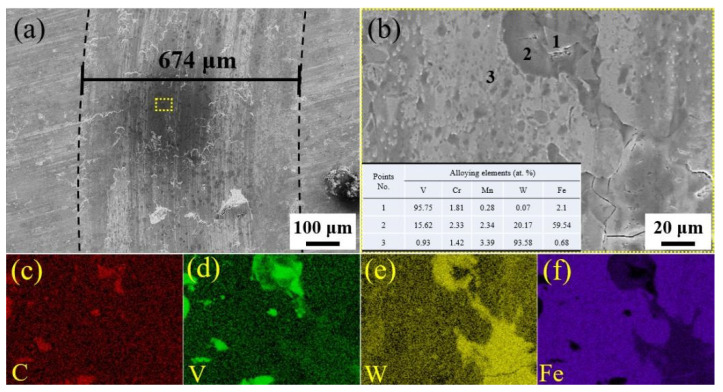
SEM morphologies and the corresponding EDS elemental distribution of wear tracks in the QPT-treated sample. (**a**) SEM image of wear track; (**b**) Magnified morphology of (**a**) in the yellow box with the dotted line; (**c**) Distribution of C element; (**d**) Distribution of V element; (**e**) Distribution of W element; (**f**) Distribution of Fe element.

Figure 5 shows that there was excellent wear resistance in the MQPT-treated sample while serious wear appeared in the QPT-treated sample. The wear mechanism of VCFC has a synergistic effect concerning the adhesive wear mechanism and the abrasive wear mechanism. However, the adhesive wear mechanism played a dominant role in the MQPT-treated sample; W-rich material was peeled from the counterpart to cover the surface of the wear track, but serious wear occurred in several channels in contact with the counterpart, as shown in Figure 9. However, the abrasive wear mechanism played a dominant role in the QPT-treated sample because of the existence of brittle M_7_C_3_ carbide, as shown in Figure 10. Some VC carbides were enclosed in brittle M_7_C_3_ carbide, as shown in Figure 8a; brittle M_7_C_3_ carbide was easily broken and peeled to form a crater around the VC particle, and the debris of the brittle M_7_C_3_ carbide was served as abrasive particles to flatten out on the surface of the wear track; therefore, serious wear occurred in the QPT-treated sample. On the other hand, metastable retained austenite played an important role in strengthening wear resistance. According to XRD patterns, a higher ratio of retained austenite existed in the MQPT-treated sample, and retained austenite transformed into martensite could reduce the stress concentration, and retained austenite could be considered as a soft phase, which could possess a higher absorbing energy to coalesce with the defects from other phases, thus hindering crack growth during the loading process [33,34]. Meanwhile, the phase transformation from retained austenite to martensite occurred and increased the hardness, thus resulting in excellent wear resistance and low wear rate [35,36,37].

For tribological behaviors of MQPT-treated sample, wear rate under wear load and wear time exhibited a downward trend in a parabola, as shown in Figure 5c,d. This could be attributed to two factors: Hard VC particles and TRIP effect. Firstly, a serious wear occurred in the initial stage because of the rough surface and low hardness. In terms of time, soft retained austenite was firstly ground out from the convex surface, and then martensite was also pushed out, thus resulting in the exposure of VC particles on the surface of the wear track. Serious wear occurred in measured material and the counterpart. Consequently, debris contained a large amount of W element from the counterpart apart from Fe and V elements of VCFC. In this stage, the retained austenite played little or no role in enhancing wear resistance because there was no time for the retained austenite to transform to bcc-structure martensite of high hardness, thus resulting in a higher wear rate and entering the stable stage. Secondly, the slope of the wear rate decreased gradually because of strain-induced work hardening and complete contact between the counterpart and measured material at the stable stage, thus resulting in excellent wear resistance because the high ratio of the bcc structure with a high hardness existed in the matrix, which was attributed to phase transformation of the retained austenite with the increase of wear load and wear time [38].

## 5. Conclusions

In this work, different heat treatments were employed to tailor the microstructure, mechanical properties and wear resistance of VCFC; meanwhile, SEM, EDS, XRD, EBSD and TEM were employed to evaluate synthetically the relationship between microstructure and mechanical properties and wear resistance of the samples under different heat treatments. The main conclusions are as follows:

(1) Metastable retained austenite with fcc structure could be stabilized at room temperature with an appropriate process of post heat treatments; the volume content of retained austenite under MQPT-treated sample had a maximum value of 25.7%, which is 56.7% higher than that of the QPT-treated sample.
(2) Morphology features of VC and M_7_C_3_ carbide changed using different heat-treated processes. Phase types did not change while the shape, size and distribution of carbide changed in QP, MQP and QPT-treated samples. However, in the MQPT-treated sample, M_7_C_3_ carbide and M_3_C disappeared, accompanied with the formation of net-like precipitates.
(3) The maximum hardness of 705 HV appeared in the QPT-treated sample accompanied with the minimum tensile strength; this was attributed to a large amount of hard VC and brittle M_7_C_3_ carbide. However, abundant retained austenite contributed to a minimum hardness of 634 HV and relatively higher tensile strength because the retained austenite possessed low hardness and good toughness to reduce the possibility of cracking during the loading process.
(4) The average hardness increased because phase transformation from retained austenite to martensite occurred during the friction-wear process, thus resulting in excellent wear resistance and lower wear rate with the increase of wear load and wear time; the wear rate of the MQPT-treated sample was only 1.83 × 10^−6^ mm^−3^/(N × m) under wear load of 20 N and wear time of 5 h when the wear rate of the QPT-treated sample was 2.64 × 10^−6^ mm^−3^/(N × m).

## Figures and Tables

**Table 1:** Heat treatment schedules of the VCFC.

Heat Treatment Process	Procedure Name			
	QP	QPT	MQP	MQPT
Mn partitioning T/t	-	-	700 °C/15 min	700 °C/15 min
Austenization T/t	1000 °C/30 min	1000 °C/30 min	1000 °C/30 min	1000 °C/30 min
Quenching T/t	100 °C/5 min	100 °C/5 min	100 °C/5 min	100 °C/5 min
C partitioning T/t	360 °C/30 min	360 °C/30 min	360 °C/30 min	360 °C/30 min
Tempering T/t	-	280 °C/120 min	-	280 °C/120 min
Cooling method	in air	in air	in air	in air

## Data Availability

The data presented in this study are available on request from the corresponding author.
